# Altered brain state dynamics between preterm and term-born infants

**DOI:** 10.1162/IMAG.a.65

**Published:** 2025-07-07

**Authors:** Srikanth R. Damera, Sudeepta Basu, Kushal Kapse, Jon Murnick, Nickie Andescavage, Catherine Limperopoulos, Josepheen De Asis-Cruz

**Affiliations:** Developing Brain Institute, Children’s National, Washington, DC, United States

**Keywords:** dynamic functional connectivity, preterm birth, neonatal fMRI, default mode network, brain states, LeiDA

## Abstract

Preterm birth alters the development of infant brain networks. However, most prior studies investigate its effects on static brain networks rather than dynamic brain states. Increasing evidence shows that brain state dynamics reflect cognitive processes beyond what is revealed by static brain networks. In the current study, we identify infant brain states and test how their dynamics are influenced by prematurity. To do so, we applied Leading Eigenvector Analysis (LEiDA) to resting-state fMRI data collected from term (n = 86) and preterm-born (n = 102) infants after term equivalent age which identified four discrete brain states across both groups. These brain states corroborate, in an independent dataset, those found in the only other large-scale study of infant brain states. Furthermore, we show that term-born infants spent more time than preterm infants in a “Transmodal State” that resembles the Default-Mode Network in adults. In contrast, preterm birth was associated with transitioning from the Transmodal state to states dominated by sensory processing or where subcortical and cortical areas were dissociated from each other. Together, these findings suggest that preterm birth alters not just static brain networks as previously shown but also brain network dynamics.

## 1 Introduction

Individuals born preterm, before 37 weeks gestational age (GA), are at higher risk of neurodevelopmental disabilities later in life (Gamarra-Oca et al., 2021; E. E. Rogers & Hintz, 2016; Woythaler, 2019). Many of these neurodevelopmental disabilities can be categorized as altered cognitive states, such as disturbed sleep (Giannotti et al., 2008; Logan & McClung, 2019; Philipsen et al., 2006; Richdale & Schreck, 2009), attention (Blaser et al., 2014; Faraone et al., 2015; Keehn et al., 2013), and emotion (Lord et al., 2020; Xiao et al., 2022; Zantinge et al., 2019). These altered states may either be a cause or a consequence of neurodevelopmental disabilities. Deviations from the *in-utero* environment due to preterm birth, such as inappropriately early sensory experience, may alter the normal development of cognitive systems and, consequently, increase the risk of future disability. Prior studies have shown that one can make inferences about cognitive states by examining the dynamic patterns of connectivity among brain areas, that is, their “brain state” (Allen et al., 2014; Ashwood et al., 2022; Baldassano et al., 2017; Yates et al., 2022). Thus, establishing if there are differences in brain states between preterm and term-born infants in the perinatal period may provide new insights into the etiology of neurodevelopmental disabilities.

Studies examining the effects of preterm birth on developing brain networks traditionally use functional magnetic resonance imaging (fMRI) data collected during sleep (Bouyssi-Kobar et al., 2019; Kwon et al., 2015; C. E. Rogers et al., 2018; Smyser et al., 2010, 2016). Brain networks are then computed as static patterns of connectivity that are stable over several minutes. Yet, by collapsing across longer timescales they ignore the dynamics of neural activity that characterize brain states (Lurie et al., 2020). In contrast, a growing number of studies in adults use a class of methods called dynamic functional connectivity (dFC) (Allen et al., 2014; Cabral et al., 2017; Iraji et al., 2020; Lurie et al., 2020; Martínez et al., 2020; Olsen et al., 2022) to examine the changing dynamics of connectivity patterns that vary over shorter (e.g., seconds) timescales. These techniques make it possible to track changes in brain states associated with sleep stage (Damaraju et al., 2020), attention (C. Wang et al., 2016; Xie et al., 2018), and anesthesia (Hutchison et al., 2014; Miao et al., 2023; Tsurugizawa & Yoshimaru, 2021), for example. Furthermore, dFC may be better at capturing behavioral variability (Liégeois et al., 2019; Vidaurre et al., 2021) in neurotypical adults and categorizing disease states (Jin et al., 2017; Rashid et al., 2016) than static FC.

Only two studies have used dFC to study the effect of preterm birth on infant brain states (França et al., 2024; Ma et al., 2020). These studies focused primarily on moderate (32 weeks ≤ GA <34 weeks) and late (34 weeks ≤ GA <37 weeks) preterm infants. Furthermore, unsupervised clustering methods used to calculate dFC states are highly sensitive to the underlying data. Therefore, we performed this study in primarily very (28 weeks ≤ GA <  32 weeks) and extremely (GA <  28 weeks) preterm infants to determine if prior results are applicable to this population. In the current work, we conducted a case-control study in which we used dFC to characterize infant brain state dynamics in preterm and term infants scanned after term-equivalent age (GA ≥37 weeks). We hypothesized that we would be able to identify brain states like those identified by França et al. We also hypothesized that these brain state dynamics would differ among preterm and term infants and that these changes would correlate with known major clinical comorbidities. Finally, we predicted that certain brain states would show maturational changes. To assess this, we first identified brain states common to term and preterm infants. We then tested if term and preterm infants differed in the amount of time they spent in each state and how they transitioned among states. Next, we examined how brain state dynamics changed with age at scan. Finally, we tested if brain state dynamics in preterm infants could be explained by common clinical co-morbidities. Overall, our results support the existence of brain states found in prior work and extend those results by finding novel effects of very and extreme preterm birth on brain state dynamics.

## 2 Methods

### 2.1 Participants

Subjects were recruited from the Neonatal Intensive Care Units (NICU) at Children’s National Hospital (CNH, Washington, DC). All infants were prospectively recruited as part of a larger longitudinal observational study investigating neurodevelopment in preterm infants. Term infants used as healthy controls were recruited from a preexisting database, and the study was approved by the institutional review board of CNH and written informed consent was obtained from parents of study participants.

All MRI studies were reviewed by a single pediatric neuroradiologist (J.M.), and preterm scans were assessed for brain injury using the Kidokoro score (Kidokoro et al., 2013). The Kidokoro score summarizes radiographic abnormalities in the cerebral white matter, cortical gray matter, deep gray matter, and cerebellum, as a global brain abnormality score. In this study, patients with Kidokoro scores ≥8, indicative of moderate-to-severe global brain injury, were excluded. Similarly, subjects with an average framewise displacement (FD) ≥0.5 mm were excluded. We also excluded infants that were either small for gestational age (SGA; <10^th^ percentile by weight) or large for gestational age (LGA; >90^th^ percentile by weight), so that birth weight and GA at birth are highly correlated (r = 0.79); thus, birth weight was excluded from the regression analyses discussed below*.* Exclusion criteria for all infants also included chromosomal anomalies, dysmorphic features, congenital brain malformations, central nervous system infection, and metabolic disorders. Finally, MRI acquisitions yielding less than 5 minutes of usable resting-state data also were excluded.

### 2.2 Clinical variables

The effects of several common co-morbidities in preterm infants, known to alter typical brain development, were examined. These included diagnosis of necrotizing enterocolitis (NEC), bronchopulmonary dysplasia (BPD), and patent ductus arteriosus (PDA). In addition to these measures, we also recorded biologic sex at birth, birth weight, and exposure to prenatal steroids (PNS). Postmenstrual age (PMA) of preterm infants at the time of MRI was derived as their birth gestational age plus their chronological age at the time of MRI. Table 1 summarizes the clinical characteristics of our study cohorts.

**Table 1.** Clinical characteristics of the cohort.

	Term infants (n = 86)	Preterm infants (n = 102)	t-statistic	(p-value)
Birth gestational age (μ ± σ weeks)[min-max]	39.5 ± 1.1 [37-41.6]	28.2 ± 3.0 [23-35.7]	36.3	p<0.001
Post-menstrual age at scan (μ ± σ weeks)[min-max]	41.9 ± 1.8 [38.6-47.3]	40.1 ± 1.9 [37-48.1]	6.8	p<0.001
Birth weight (μ ± σ grams)[min-max]	3307.4 ± 425.0 [2280-4184]	1049.2 ± 470.4 [481-2600]	35	p<0.001
Sex (n)	46M	47M		
Kidokoro score (μ ± σ score)[min-max]	-	3.2 ± 1.9 [0-7]		
Bronchopulmonary dysplasia (n)	-	27		
Necrotizing enterocolitis (n)	-	27		
Patent ductus arteriosus present (n)	-	50		
Prenatal steroids given (n)	-	59		

### 2.3 Functional MRI data acquisition

Preterm and full-term infants were scanned after term-equivalent age using a 3 Tesla MRI scanner (Discovery MR750, General Electric Medical, Systems-Waukesha, WI) with an 8-channel receiver head coil. To facilitate sleep and minimize in-scanner movement, neonates were fed, swaddled in a warm blanket, and immobilized using an infant vacuum pillow. To protect their hearing from scanner noise, they wore silicone earplugs and adhesive earmuffs. Anatomical T2-weighted fast spin echo (3D Cube) data were obtained in both full-term and preterm infants with a mean voxel size = 0.625 x 1 x 0.625 mm^3^ [range: 0.2539 X 1.0 X 0.2539 – 0.7301 X 1.0 X 0.7301 mm^3^]. Resting-state data, in full-term and preterm infants, were collected using a T2*-weighted gradient-echo planar imaging (EPI) sequence: TR = 2000 ms, TE = 35 ms, mean voxel size = 2.954 X 2.954 X 3.078 mm^3^ [range: 1.875 X 1.875 X 3 – 3.125 X 3.125 X 3 mm^3^], flip angle = 60°, slice number = 29 to 41, and number of volumes = 200-300.

### 2.4 Functional MRI data pre-processing

Image preprocessing was performed using AFNI (Cox, 1996) and custom MATLAB scripts. First, within-volume motion correction was performed on EPI data and then anatomical and EPI volumes were moved to the same space by aligning their centers. The first four acquisitions of each run were then discarded to allow for T1 stabilization. The data were further preprocessed by 1) applying slice-time correction, 2) despiking EPI images to mitigate the effect of BOLD signal outliers on image registration and improve motion regressor estimation (Jo et al., 2013), 3) applying bias-field correction, 4) realigning all EPI images to the volume to the base image (i.e., the image with the least number of outliers), 5) resampling all EPI images to a 3 mm isotropic grid, 6) co-registering the base image to the anatomical scan, 7) segmenting anatomical images to produce gray matter (GM), white matter (WM), and cerebrospinal fluid (CSF) tissue masks using DrawEM (Makropoulos et al., 2014), and 8) non-linear normalization of all images including tissue masks to the Shi neonatal template brain (Shi et al., 2011). Transformations in steps 4, 6, and 8 were applied simultaneously to EPI BOLD data. The resulting resting state data were then intensity scaled to a global mode of 1000 and then bandpass filtered between 0.02 Hz–0.1 Hz. Data were then cleaned by removing the effect of tissue and motion regressors. Tissue regressors were derived from the eroded and localized WM and the first five principal components of the averaged CSF signal. Motion regressors included linearly demeaned translational and rotational head motion parameters (generated in step 4), their temporal derivatives, and quadratic terms.

### 2.5 Computing dynamic functional connectivity (dFC) matrices

**Fig. 1.**
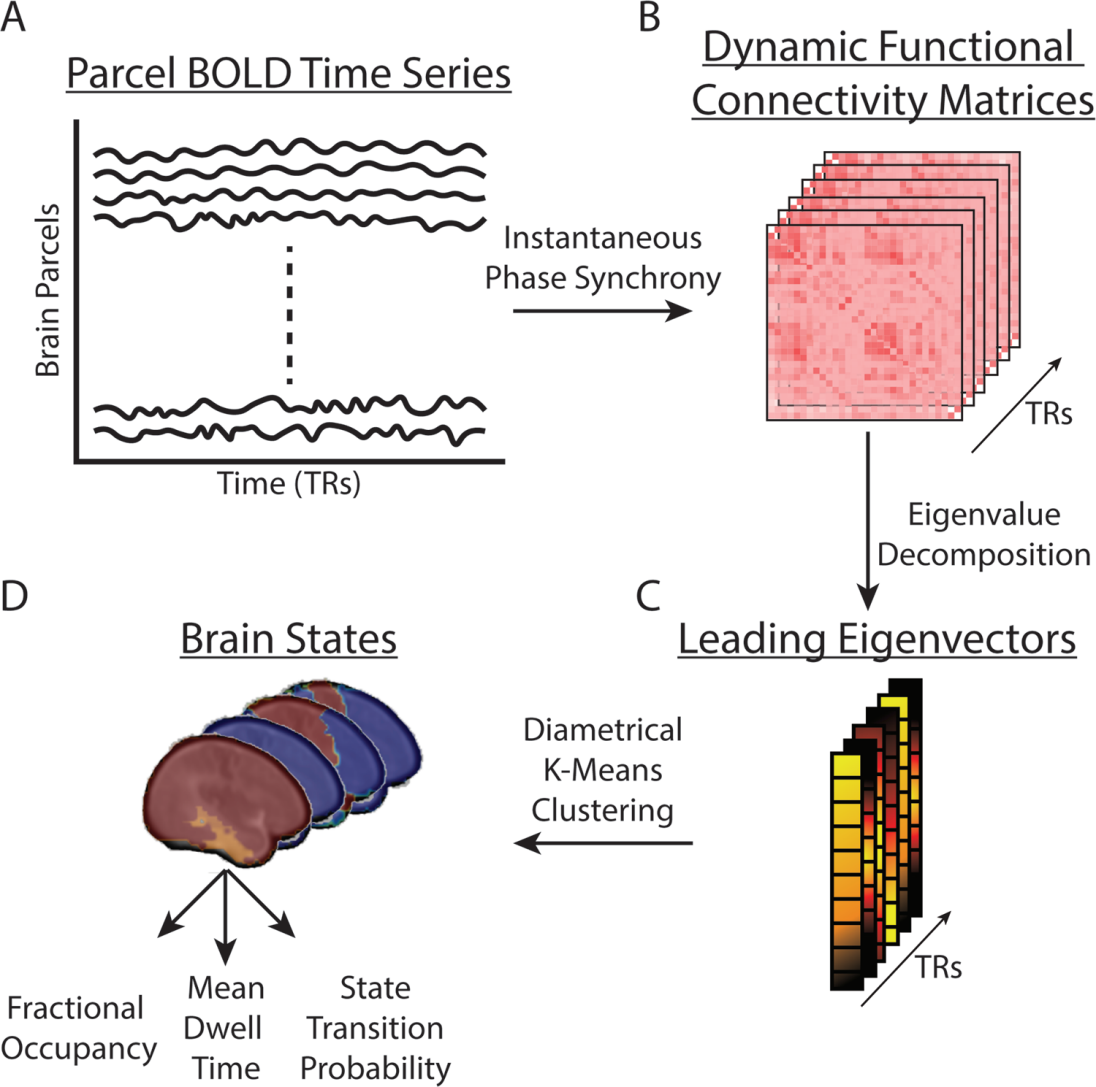
Analysis Overview. (A) BOLD fMRI data are first averaged into a set of parcels defined by the AAL atlas. (B) Instantaneous phase synchrony is computed to define Parcel-by-Parcel functional connectivity matrices at each TR. (C) Eigenvalue decomposition is performed for each matrix, and the leading eigenvector corresponding to the largest eigenvalue is extracted at every TR. The sign of values in the leading eigenvector can be used to partition brain parcels into communities. (D) Diametrical K-means clustering is used to group leading eigenvectors into brain states. Three main statistics: Fractional Occupancy (FO), Mean Dwell Time (DT), and State Transition Probability (TP) can be computed to characterize brain state dynamics.

Dynamic functional connectivity was computed at each TR as the instantaneous phase coherence (Cabral et al., 2017) among 93 brain regions defined by the infant AAL atlas (Shi et al., 2011) parcels. First, we averaged unsmoothed BOLD timeseries data across all voxels within each parcel (Fig. 1A). Then, we demeaned and detrended the parcel-level timeseries data. Next, we computed the Hilbert transform and extracted instantaneous phase values at each TR. Finally, phase coherence between any two parcels at a given TR was defined as the cosine of the phase difference between them. This procedure resulted in a Parcel-by-Parcel-by-TR dFC matrix (Fig. 1B).

### 2.6 Leading eigenvector dynamic analysis (LEiDA)

**Fig. 2.**
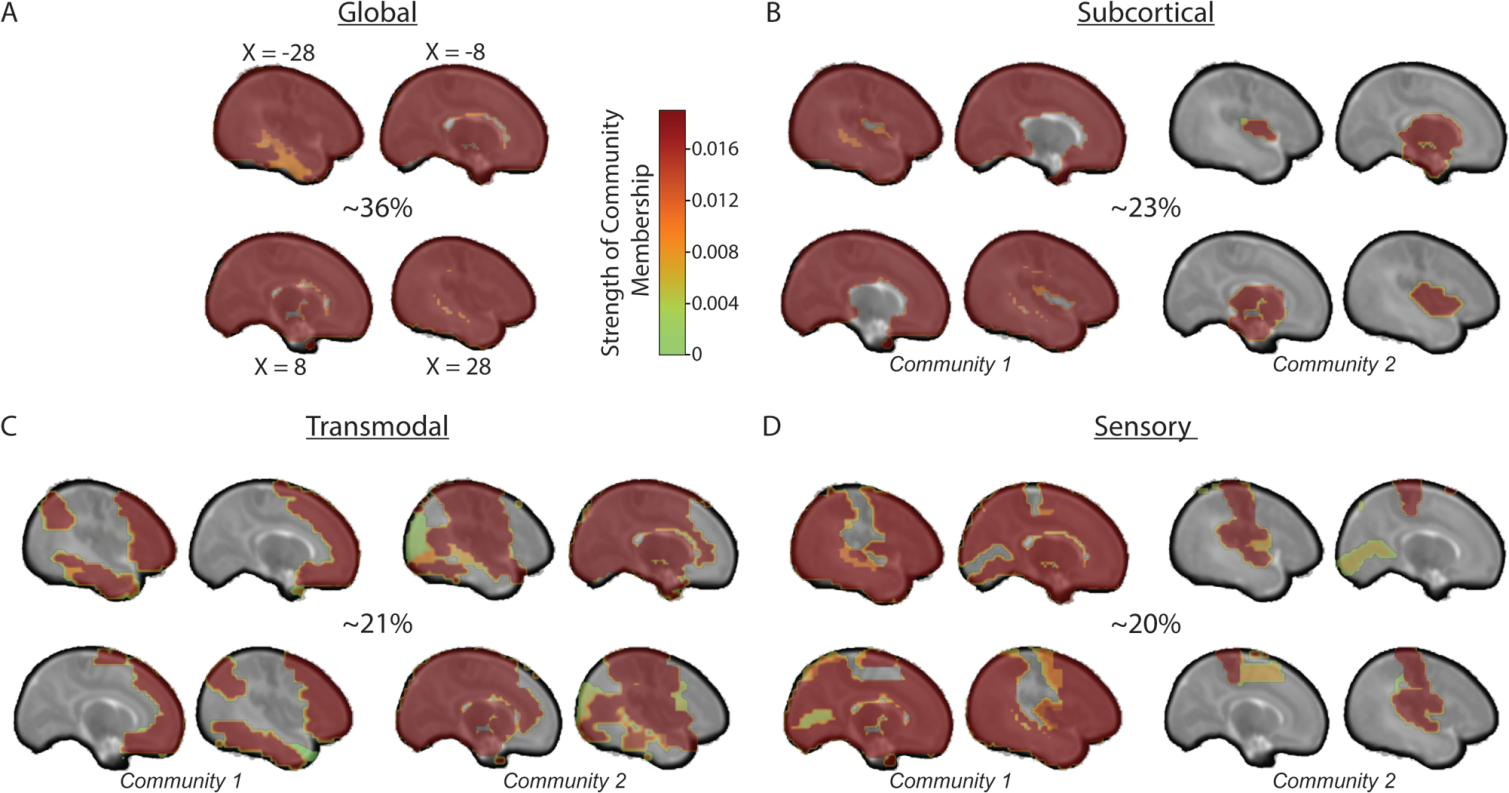
Characterizing Neonatal Brain States. LEiDA identifies four brain states across both preterm and term-born infants. States from panels (A-D) Global, Subcortical, Transmodal, and Sensory. Each state except the Global State was able to be partitioned into two communities. Numbers in the center of each panel show the fractional occupancy of infants in each state across both preterm and term infants. Colors reflect the strength of a parcel’s affiliation with its community. X-values refer to slice position in the brain-volume.

We applied LEiDA (Cabral et al., 2017; Martínez et al., 2020; Newman, 2006) to the dFC matrix to continuously track each infant’s brain states. First, at each TR, the Parcel-by-Parcel adjacency matrix is summarized by its leading eigenvector, that is, the eigenvector associated with the largest positive eigenvalue. The sign (+/-) of the leading eigenvector’s elements separates brain areas into at most two communities while their magnitude specifies the strength with which a brain area belongs to its community (Newman, 2006). These leading eigenvectors were pooled across subjects and were clustered into distinct clusters using diametrical K-means clustering (Fig. 1D) (Dhillon et al., 2003). The number of clusters (k = 4) was selected by identifying the elbow in the within sum-squared error plot. Finally, brain states were defined as the cluster centroids, each comprising either one or two communities based on the sign of centroid elements (Fig. 2) (Newman, 2006).

### 2.7 Characterizing brain state dynamics

Three main measures were computed to capture brain state dynamics (Fig. 1D) (Allen et al., 2014; Iraji et al., 2020; Xie et al., 2018). The first was Fractional Occupancy which captures the fraction of the total time an individual is in any one state. The second was Mean Dwell Time which captures how long an individual continuously stays in each brain state. Finally, we computed state Transition Probabilities which captures the probability of transitioning between any two states. For two states, State 1 and 2, it is calculated as the number of times an individual leaves State 1 and enters State 2 divided by the total number of times an individual leaves State 1. All metrics were calculated without censoring volumes to avoid introducing any temporal discontinuities.

### 2.8 Fitting linear models to brain state dynamics

Several linear regression models were used to test the effect of preterm birth and PMA on brain state dynamics while controlling for nuisance variables. These models were fit using the *lm* (Bates et al., 2015) functions in *R*. In these models, PretermBirth , Sex , and ClinicalCovariates were categorical variables while PMA , and Motion , were continuous variables. The clinical covariates included diagnosis of NEC, BPD, PDA were made, and if prenatal steroids (PNS) were received and were coded as binary variables. Motion was defined as the average FD per infant. We first tested the effect of preterm birth on brain state dynamics using the following linear regression model:

$$\begin{array}{l} Brain\;State\;Dynamics\;\sim \;1+PretermBirth\\ \;\;\;+PMA+Motion+Sex \end{array}$$ [Model 1]

We also tested if, in preterm infants, clinical comorbidities associated with preterm birth predicted brain state dynamics. We did this using the following model:

$$\begin{array}{l} Brain\;State\;Dynamics\;\;\sim \;1+BirthGA+PMA\\ \;\;\;+Motion+Sex+BPD+NEC+PDA+PNS \end{array}$$ [Model 2]

### 2.9 Statistical analysis

For Models 1 and 2, the p-values for each factor (e.g., PretermBirth ) were Hommel-corrected for multiple comparisons across ROIs. Hommel-corrected p-values (p_HC_) are reported throughout the text, but uncorrected p-values are reported in the tables.

## 3 Results

### 3.1 Clinical and imaging characteristics of the study cohort

In total, 266 preterm and 212 term-born infants were scanned after term-equivalent age after excluding those who were SGA, LGA, or had chromosomal anomalies, dysmorphic features, congenital brain malformations, central nervous system infection, and metabolic disorders. For term-born infants, 86 infants had at least 5 minutes of usable data. For the preterm group, 182 infants had a kidokoro score <8; of those, 107 had at least 5 minutes of usable data, and 102 had an average FD < 0.5. The final sample included data from 86 term-born infants (μ_GA_ ± σ_GA_ = 39.5 ± 1.1; μ ± σ_CA _= 41.9 ± 1.8 weeks; μ ± σ_Kidokoro_ = 3.2 ± 1.9 weeks; 46 males) and 102 preterm (μ_GA_ ± σ_GA_ = 28.2 ± 3.0; μ_PMA_ ± σ_PMA_ = 40.1 ± 1.9 weeks; 47 males) infants (Table 1). Of the 102 preterm infants, 27 were diagnosed with BPD, 27 were diagnosed with NEC, 50 had a PDA, and 59 received prenatal steroids.

### 3.2 Characterizing neonatal brain states

We identified four brain states across both preterm and term-born infants (Fig. 2). In the Global state (Fig. 2A), there is only a single community that includes all brain areas. In the Subcortical state (Fig. 2B), subcortical and cortical areas belong to separate communities. In the Transmodal state (Fig. 2C), frontal, inferior parietal, and inferior temporal areas belong to the same community. In the Sensory state (Fig. 2D), primary sensory areas, except the right primary visual cortex, belong to the same community. Notably, the left primary visual cortex unlike auditory and somatosensory cortices is only a weak participant in the community. Infants spent the most time in the Global state and roughly an equal amount of the time in the remaining three states.

### 3.3 Preterm birth alters fractional occupancy and mean dwell time

**Fig. 3.**
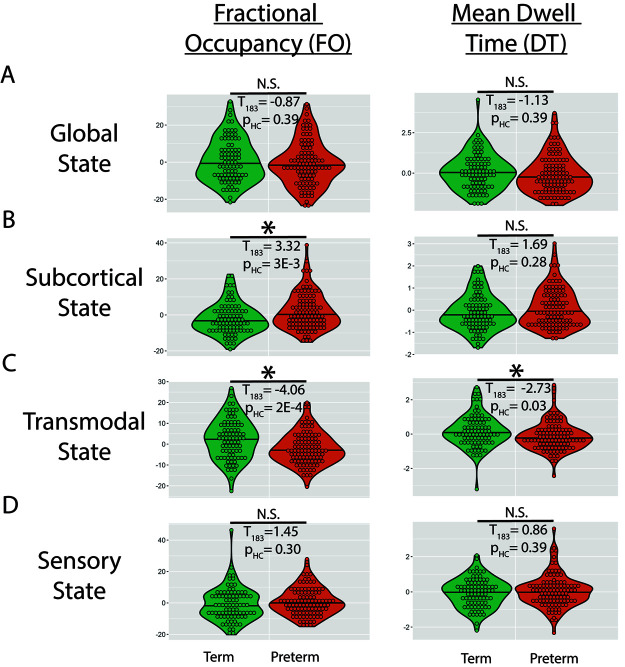
The Effect of Preterm Birth on Fractional Occupancy and Mean Dwell Time. (A) Partial dependency plots examining the effect of preterm birth on fractional occupancy and mean dwell time in the Global State after controlling for the remaining regressors. *Indicates p_HC_ < 0.05 familywise error. (B-D) Same as (A) but for the remaining brain states.

We first tested how preterm birth altered Fractional Occupancy (Table S1) and Mean Dwell Time (Table S2) in each of the four states (Fig. 3; Model 1). Preterm birth was associated with a significant increase in the Fractional Occupancy (T_183_ = 3.32; p_HC_ = 0.003) of the Subcortical state, and a significant decrease in the Fractional Occupancy (T_183_ = -4.06; p_HC_ = 0.0002) as well as the Mean Dwell Time (T_183_ = -2.73; p_HC_ = 0.028) of the Transmodal state.

### 3.4 PMA alters fractional occupancy and mean dwell time

**Fig. 4.**
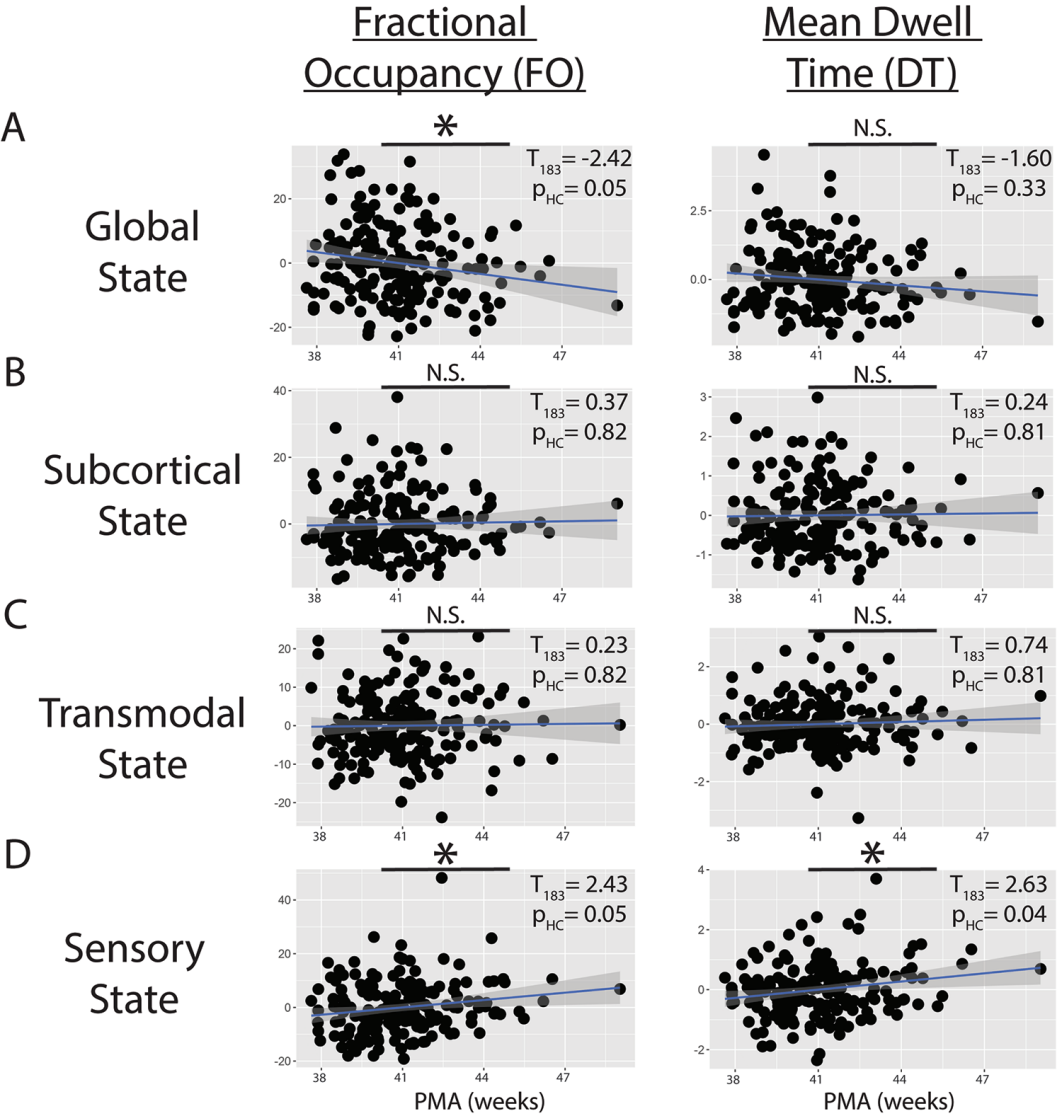
The Effect of PMA on Fractional Occupancy and Mean Dwell Time. (A) Partial dependency plots examining the effect of PMA on fractional occupancy and mean dwell time in the Global State after controlling for the remaining regressors. Line in scatter plots reflects the regression line. *Indicates p_HC_ < 0.05 familywise error. (B-D) Same as (A) but for the remaining brain states.

Next, we tested how PMA altered Fractional Occupancy and Mean Dwell Time in each of the four states (Fig. 4). For the Global State (Fig. 4A; Model 1), PMA at scan had a significant negative effect on Fractional Occupancy (T_183_ = -2.42; p_HC_ = 0.050), and a significant positive effect on both Fractional Occupancy (T_183_ = 2.43; p_HC_ = 0.048) and Mean Dwell Time (T_183_ = 2.63; p_HC_ = 0.037) for the Sensory State (Fig. 4D).

### 3.5 Preterm birth alters transitions among states

**Fig. 5.**
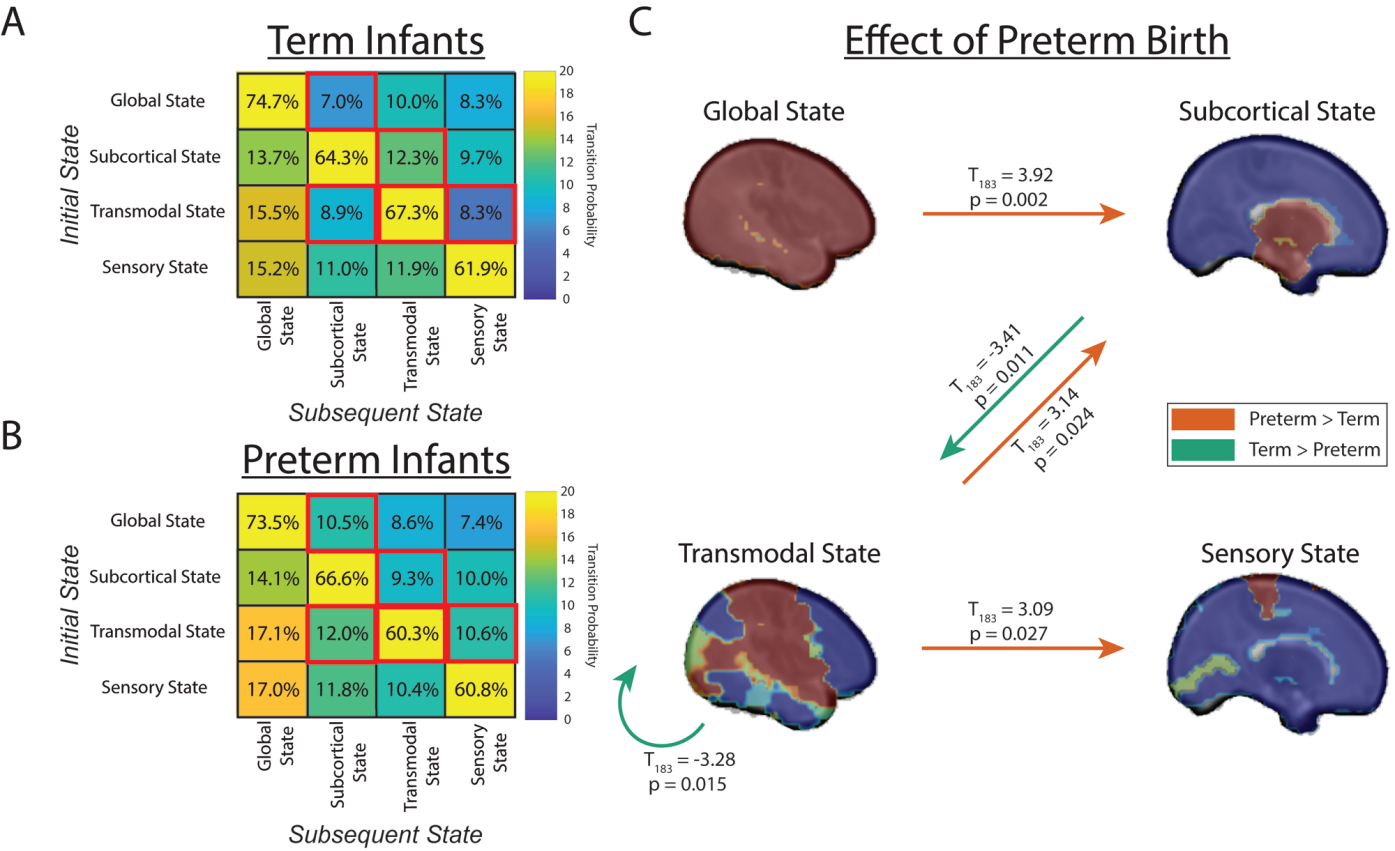
Preterm Birth Alters Transitions Among States. The Transition Probability of moving among states for term infants is depicted in Panel (A) and for preterm infants in Panel (B). Colors reflect the Transition Probability of moving from an initial state (rows) to the subsequent state (column). (C) Shows significant differences (p_HC_ ß< 0.05) in the Transition Probability between term and preterm infants. Orange arrows reflect a greater probability of transitioning states in preterm infants, and green arrows reflect a greater probability of transitioning states in term infants.

We then tested how preterm birth and PMA altered transitions among the four brain states (Fig. 5; Model 1). This revealed no significant effect of PMA on the probability of transitioning between states. Preterm Birth was associated with greater rates of transition from the Global to the Subcortical State (T_183_ = 3.92; p_HC_ = 0.002), from the Transmodal to the Subcortical State (T_183_ = 3.14; p_HC_ = 0.024), and from the Transmodal to the Sensory State (T_183_ = 3.09; p_HC_ = 0.027). In contrast, Term Birth was associated with greater rates of transition from the Subcortical to the Transmodal State (T_183_ = -3.41; p_HC_ = 0.011) and higher duration in the Transmodal State (T_183_ = -3.28; p_HC_ = 0.015).

### 3.6 Clinical comorbidities are not associated with brain state dynamics in preterm infants

Preterm birth is highly correlated with clinical comorbidities that may later brain development and brain state dynamics. Thus, we tested if, for preterm infants, there was an association between several of these comorbidities and brain state dynamics (Model 2). Our results showed no significant association between any variable and either Factional Occupancy (Table S3) or Mean Dwell Time (Table S4).

### 3.7 Gestational age at birth but not PMA is associated with scanner motion

We tested if PMA and gestational age at birth were associated with scanner motion. We found that there was a significant negative association between gestational age at birth and scanner motion (T_185_ = -2.42, p = 0.0165). However, there was no relationship between PMA and scanner motion (T_185_ = -0.43, p = 0.67). We repeated our analyses using more stringent criteria—FD threshold of 0.3—and removing subjects that had >20% outliers (Smith et al., 2022). As a result of these choices, 0 Term and 14 preterm subjects were removed from the analyses and there was no longer a significant association between gestational age at birth and motion (T_176_ = -1.63, p = 0.10). Using these more restricted data, we were able to recover the same four brain states and regression analyses showed the same significant effects (Tables S5-S6).

## 4 Discussion

In the current study, we used dFC methods to determine infant brain state dynamics. We showed that infant brain activity across both groups can be summarized by four distinct brain states that we termed: Global, Subcortical, Transmodal, and Sensory. Preterm birth reduces the amount of time spent in the Transmodal State and increases the amount of time spent in the Sensory and Subcortical States. We also showed that, with advancing PMA, all infants spend less time in the Global State. Together, these results show that infant brain dynamics are altered by preterm birth, which may play an important role in shaping emerging brain networks and neurodevelopmental outcomes.

The current work is an important validation and extension of the only other large-scale study of neonatal brain-states (França et al., 2024). Like França et al., we identified states that highlight global synchrony among all brain areas, synchrony among somatosensory areas, and synchrony among transmodal areas. We also recovered these states despite using different scanning parameters and a more balanced cohort of term and preterm infants. In addition to França et al., only one other study, Ma et al. (2020), has investigated the effect of preterm birth on neonatal brain dynamics. However, this study computed dFC between ICA-derived functional networks rather than anatomical regions-of-interest. This and other methodological differences make it difficult to compare their brain-states and descriptions of brain-state dynamics to our work. Collectively, these results strengthen the observations that the global, somatosensory, and default mode dominated states reflect true brain states that characterize neonatal brain dynamics.

The Sensory State highlights early but graded interactions among the sensory cortices. Neonates spend a greater fraction of their time in this state as they age which may reflect the joint maturation the sensory areas. However, somatosensory and auditory cortices show stronger community membership than the visual cortex in this state. This suggests that the auditory and somatosensory cortices are more synchronous with each other and segregated from the rest of the brain than the visual cortex. This strong early interaction between somatosensory and auditory cortices may be crucial for future language development (Dall’Orso et al., 2020; Rauschecker, 2011). Similar results are seen in recent studies of early brain organization which shows that somatosensory and auditory areas develop connectivity profiles distinct from the rest of the cortex over the third trimester (Xia et al., 2024). In contrast, the connectivity profile of the visual cortex seems more like other cortical areas. Theories of brain organization suggest that early sensory cortex development helps organize higher-order brain areas which is important for linking perceptual and cognitive processing (Arcaro & Livingstone, 2024; Groen et al., 2022; Knapen, 2021; Steel et al., 2024). Future studies should investigate how differences between brain-wide connectivity among the sensory systems drive the organization of higher-order brain areas and behavior.

It is interesting to note the strong negative association between motion and time spent in the Sensory State. One possibility is that it reflects negative association between birth GA and motion; however, inclusion or exclusion of birth GA in our model does not appreciably change the effect of motion. Furthermore, there is low multicollinearity between birth GA and motion. Alternatively, motion may be associated with other physiological variables such as sleep state. A recent study shows that scanner head motion in AS trends higher than QS although there was no statistically significant difference (Mueller et al., 2025). As infants get older, they spend progressively more time in quiet than active sleep. If the Sensory State is more easily recovered during quiet sleep, then that may explain both its negative association with head motion and positive association with increasing GA. This further highlights the importance of accounting for sleep state in future studies.

The Transmodal State identified in this study appears to be a rudimentary form of the adult Default Mode Network (DMN) (Andrews-Hanna et al., 2010, 2014). In particular, the topography of this brain state is like the dorsal medial subsystem of the DMN. In adults, this subsystem is thought to be involved in mentalizing—the process of inferring beliefs about oneself or others (Andrews-Hanna et al., 2014). In newborns, this system may be involved in tracking their internal affective state as they begin to interact with the world. Intriguingly, we find that preterm birth reduces the amount of time infants spend in this state and favors more time in other states such as the Sensory State. We speculate that preterm birth biases infants toward processing sensory information at the cost of integrating this information with their internal state. If true, this may explain why preterm infants are at higher risk of developing sensory processing disorders which are characterized by a deficit in integrating sensory information to guide behavior (Rahkonen et al., 2015; Ryckman et al., 2017; Wickremasinghe et al., 2013).

We found that preterm birth is associated with spending longer in and transitioning more frequently to the Subcortical State, which is characterized by a functional dissociation between subcortical and cortical areas. Several studies have identified a similar brain state (Abrol et al., 2017; Allen et al., 2014; Cabral et al., 2017; Martínez et al., 2020; Stevner et al., 2019). Allen et al., (Abrol et al., 2017; Allen et al., 2014; Cabral et al., 2017; Martínez et al., 2020; Stevner et al., 2019) initially hypothesized that this state may be related to drowsiness as the transition from wakefulness to sleep is characterized by increased subcortical connectivity and reduced thalamocortical connectivity. Stevner et al. (Abrol et al., 2017; Allen et al., 2014; Cabral et al., 2017; Martínez et al., 2020; Stevner et al., 2019) grouped brain states by sleep stage and found that states characterized by a dissociation between subcortical and cortical areas best corresponded to the N1 stage of sleep which marks the sleep-wake transition. Although it is unclear how adult sleep stages map onto infant sleep, the greater time preterm infants spend in the Subcortical State may be related to effects of preterm birth on sleep architecture (Uchitel et al., 2022). Alternatively, this dissociation may be due to altered thalamocortical connectivity which is known to be affected by preterm birth (Ball et al., 2013, 2015; Toulmin et al., 2021). Future studies should test if this brain state is replicable in an independent data set and then test how time spent in this state relates other physiological variables (e.g., sleep state, thalamocortical connectivity, etc.).

Both term and preterm infants were predominantly in the Global State, which is marked by a high synchronicity among all brain areas. This global signal can be found from the fetal to the adult period and likely represents a mixture of non-neuronal and neuronal sources (Gu et al., 2021; Kim et al., 2022; Liu et al., 2017; Murphy et al., 2009; Raut et al., 2021; Schölvinck et al., 2010; Turchi et al., 2018). Here, we found that the amount of time infants stayed in the Global State decreased with PMA. This is supported by work in fetuses which shows a negative association between the strength of global activity and fetal age (Kim et al., 2022). Furthermore, several studies have linked global activity with changes to neurotransmitter activity, structural changes, altered cognitive states, and disease (Kong et al., 2021; Shine, 2019; Turchi et al., 2018; P. Wang et al., 2019). A recent study in adults found that periods of global cortical synchrony were associated with mind-blanking or “local sleepiness” and reduced global cortical activity (Mortaheb et al., 2022). Interestingly, as infants age, they spend less time in active sleep which is characterized by low voltage synchronous activity (Dereymaeker et al., 2017). At this stage, while there is clearly a maturational effect on Global State, it remains unclear which of these factors contribute to this effect and how changes in the Global State are related to future risk for neurodevelopmental disability.

The exact mechanisms by which preterm birth alters brain-state dynamics are unclear. It may be due to early exposure to the *ex-utero* sensory environment and or disruption of typical development. It may also be due to clinical co-morbidities associated with prematurity. However, in the current work we did not find any association between our clinical variables and brain-state dynamics in preterm infants. It may be that our study was not powered to detect these associations or that different unmeasured variables account for the differences. Alternatively, preterm birth may be the most important factor causing changes in brain dynamics, and other factors are not as important. França et al. found that increased time in the somatomotor state, which resembles our Sensory State, was associated with higher risk of autism (França et al., 2024). As discussed above, this fits with our own results that preterm birth biases infants away from the Transmodal to the Sensory Sate. It will be important to investigate potential associations between brain-state dynamics and subsequent neurodevelopmental outcomes (beyond autism). These studies are currently underway.

There are limitations to the current study. First, like most infant MRI studies, our data were collected while infants slept, so we were unable to measure infant arousal nor sleep state. It is known that infants cycle through periods of wakefulness, alert sleep, and quiet sleep (Uchitel et al., 2022; Vanhatalo & Kaila, 2006), all of which are associated with distinct patterns of brain connectivity and likely exhibit different brain state dynamics (Tokariev et al., 2019). Similarly, work in adults has shown that inferred brain states vary between sleep and awake periods (Damaraju et al., 2020). Indeed, Damaraju et al. found that the brain states derived from the fMRI data of sleeping participants primarily tracked the different sleep states. As discussed above, this complicates the interpretation of our findings since brain states and their dynamics here are likely influenced by active versus quiet sleep states. Future studies should use simultaneous infant EEG and MRI to relate sleep states and other physiological variables (respirations, heart rate, etc.) with brain states derived from fMRI. More generally, it is an open question how imaging findings translate between awake and asleep infants (Yates et al., 2023; Zhang et al., 2019). A second limitation of the current work is that we are unable to show a correspondence between differences in brain states and outcomes due to limited neurodevelopmental data. We are currently expanding our neurodevelopmental dataset, but future studies should adopt longitudinal designs with comprehensive phenotyping to robustly link brain-state dynamics to clinical and subclinical outcomes. Finally, our study is that we only used 5 minutes of usable data per subject. This duration may limit our power to identify brain states as well as differences between preterm and term-born infants as infants may be unobserved brain-states.

In summary, we show that the dynamics of infant brain activity are affected by very extreme preterm birth. Specifically, preterm birth is associated with less time in adult-like brain states associated with mentalizing. Instead, infants may spend more time in states associated with sensory processing and those less efficient at relaying information between cortical and cortical structures. These early differences in information processing may predispose infants to atypical development of functional brain networks and, thus, neurodevelopmental disabilities.

## Supplementary Material

Supplementary Material

## Data Availability

The data that support the findings of this study are available from the lead contact, Catherine Limperopoulos, upon reasonable request. All original code has been deposited at https://github.com/srd49/DBI/tree/main/imagNeuro and is publicly available as of the date of publication. DOIs are listed in the key resources table. Any additional information required to reanalyze the data reported in this paper is available from the lead contact upon request.
