# Supplementary material for: Altered brain state dynamics between preterm and term-born infants
Source: Imaging Neurosci (Camb). 2025 Jul 7;3:IMAG.a.65. doi: 10.1162/IMAG.a.65 (PMC12330851; doi:10.1162/IMAG.a.65)
Supplement: Supplementary Material [file IMAG.a.65_supp.pdf]

Table S1 – Effects of Preterm Birth and PMA on Fractional Occupancy in Brain States

	Global State		Subcortical State		Transmodal State		Sensory State	
Predictors	$ß_{std}$	$p_{uncorrected}$	$ß_{std}$	$p_{uncorrected}$	$ß_{std}$	$p_{uncorrected}$	$ß_{std}$	$p_{uncorrected}$
Intercept	-	**0.00004*****	-	0.22	-	0.28	-	0.41
PMA	-0.19	**0.017***	0.03	0.72	0.02	0.82	0.19	**0.016***
Preterm Birth	-0.07	0.39	0.27	**0.001****	-0.32	**0.00007*****	0.11	0.15
Motion	0.11	0.15	0.06	0.39	0.06	0.37	-0.25	**0.0007*****
Sex [M]	-0.00	1	0.05	0.49	0.05	0.45	-0.09	0.18
N_{term}, $N_{preterm}$	86, 102		86, 102		86, 102		86, 102	
R^2 / R^2 adjusted	0.044 / 0.023		0.078 / 0.057		0.109 / 0.090		0.104 / 0.084	

** p<0.05   ** p<0.01   *** p<0.001*

Table S2 – Effects of Preterm Birth and PMA on Mean Dwell Time in Brain States

	Global State		Subcortical State		Transmodal State		Sensory State	
Predictors	$ß_{std}$	$p_{uncorrected}$	$ß_{std}$	$p_{uncorrected}$	$ß_{std}$	$p_{uncorrected}$	$ß_{std}$	$p_{uncorrected}$
Intercept	-	**0.0001*****	2.21	**0.03***	1.32	0.19	-0.21	0.84
PMA	-0.13	0.11	0.02	0.81	0.06	0.46	0.20	**0.009****
Preterm Birth	-0.09	0.26	0.14	0.09	-0.22	**0.007****	0.07	0.39
Motion	-0.10	0.17	-0.10	0.17	-0.15	**0.04***	-0.3	**0.00003*****
Sex [M]	0.00	1	0.02	0.74	0.03	0.65	-0.15	**0.03***
N_{term}, $N_{preterm}$	86, 102		86, 102		86, 102		86, 102	
R^2 / R^2 adjusted	0.027 / 0.005		0.022 / 0.001		0.102 / 0.082		0.160 / 0.141	

** p<0.05   ** p<0.01   *** p<0.001*

Table S3 – Effects of Clinical Variables on Fractional Occupancy in Brain States

	Global State		Subcortical State		Transmodal State		Sensory State	
Predictors	*t*	*p**uncorrected*	*t*	*puncorrected*	*t*	*puncorrected*	*t*	*puncorrected*
Intercept	2.41	**0.02***	0.85	0.40	0.73	0.47	-0.36	0.72
PMA	-1.41	0.16	0.14	0.89	0.59	0.56	1.26	0.21
Birth GA	-0.01	0.99	0.02	0.99	-0.63	0.53	0.55	0.58
Motion	0.61	0.55	1.00	0.32	0.48	0.63	-2.43	**0.02**
Sex [M]	-0.19	0.85	-0.08	0.94	0.87	0.39	-0.42	0.68
NEC [Yes]	0.94	0.35	0.06	0.95	-0.86	0.39	-0.62	0.54
BPD [Yes]	-0.31	0.76	0.36	0.72	-0.02	0.99	0.03	0.98
PDA [Yes]	0.88	0.38	-1.04	0.30	0.18	0.86	-0.16	0.87
PNS [Yes]	-0.68	0.50	-0.25	0.80	-0.13	0.90	1.35	0.18
N	102		102		102		102	
R^2 / R^2 adjusted	0.060 / -0.021		0.027 / -0.057		0.077 / -0.003		0.191 / 0.121	

* *p<0.05* ** *p<0.01* *** *p<0.001*

Table S4 – Effects of Clinical Variables on Mean Dwell Time in Brain States

	Global State		Subcortical State		Transmodal State		Sensory State	
Predictors	*t*	*puncorrected*	*t*	*puncorrected*	*t*	*puncorrected*	*t*	*puncorrected*
Intercept	2.00	**0.05***	1.08	0.28	0.04	0.97	0.28	0.78
PMA	-0.79	0.43	0.24	0.81	1.56	0.12	0.87	0.39
Birth GA	0.26	0.80	0.54	0.59	-0.01	0.99	0.75	0.46
Motion	-1.63	0.11	-0.65	0.52	-1.77	0.80	-3.23	**0.002****
Sex [M]	0.07	0.95	-0.21	0.83	0.17	0.87	-1.49	0.14
NEC [Yes]	1.22	0.23	-0.29	0.77	-1.20	0.23	-0.05	0.96
BPD [Yes]	-0.67	0.51	0.42	0.68	0.39	0.70	0.61	0.54
PDA [Yes]	0.79	0.43	-0.79	0.43	0.07	0.95	-0.73	0.47
PNS [Yes]	-0.19	0.85	-0.48	0.64	0.16	0.87	1.74	0.09

N	102	102	102	102
R^2 / R^2 adjusted	0.060 / -0.021	0.027 / -0.057	0.077 / -0.003	0.191 / 0.121

** p<0.05   ** p<0.01   *** p<0.001*

Table S5 – Effects of Preterm Birth and PMA on Fractional Occupancy in Brain States After Stringent Motion Correction

	Global State		Subcortical State		Transmodal State		Sensory State	
Predictors	*ß_{std}*	*p_{uncorrected}*	*ß_{std}*	*p_{uncorrected}*	*ß_{std}*	*p_{uncorrected}*	*ß_{std}*	*p_{uncorrected}*
Intercept	-	**0.00003*****	-	0.26	-	0.18	-	0.14
PMA	-0.22	0.008**	0.01	0.93	0.02	0.83	0.24	0.002**
Preterm Birth	-0.07	0.41	0.25	0.003**	-0.31	0.0001***	0.14	0.09
Motion	0.18	0.02*	0.01	0.91	0.10	0.18	-0.32	**0.00001*****
Sex [M]	0.01	0.92	0.06	0.46	0.05	0.48	-0.11	0.13
N_{term}, $N_{preterm}$	86, 88		86, 88		86, 88		86, 88	
R^2 / R^2 adjusted	0.073 / 0.051		0.063 / 0.041		0.112 / 0.091		0.166 / 0.147	

** p<0.05   ** p<0.01   *** p<0.001*

Table S6 – Effects of Preterm Birth and PMA on Mean Dwell Time in Brain States After Stringent Motion Correction

	Global State		Subcortical State		Transmodal State		Sensory State	
Predictors	$ß_{std}$	$p_{uncorrected}$	$ß_{std}$	$p_{uncorrected}$	$ß_{std}$	$p_{uncorrected}$	$ß_{std}$	$p_{uncorrected}$
Intercept	-	**0.0001*****	-	**0.03***	-	0.28	-	0.28
PMA	-0.15	0.07	0.09	0.98	0.27	0.266	0.20	0. 0004***
Preterm Birth	-0.05	0.57	-0.19	0.11	0.13	0.024*	0.07	0.10
Motion	-0.01	0.87	-0.08	0.12	-0.34	0.277	-0.3	0.000001***
Sex [M]	0.02	0.76	0.02	0.96	-0.19	0.759	-0.15	0.01*
N_{term}, $N_{preterm}$	86, 88		86, 88		86, 88		86, 88	
R^2 / R^2 adjusted	0.020 / -0.002		0.028 / 0.006		0.069 / 0.048		0.3/ 0.141	

* p<0.05   ** p<0.01   *** p<0.001
